# Efficacy of Caffeine Treatment for Wood Protection—Influence of Wood and Fungi Species

**DOI:** 10.3390/polym13213758

**Published:** 2021-10-30

**Authors:** Miloš Pánek, Vlastimil Borůvka, Jana Nábělková, Kristýna Šimůnková, Aleš Zeidler, David Novák, Robert Černý, Klára Kobetičová

**Affiliations:** 1 Department of Wood Processing and Biomaterials, Faculty of Forestry and Wood Sciences, Czech University of Life Sciences, Kamýcká 129, 165 00 Prague, Czech Republic; boruvkav@fld.czu.cz (V.B.); simunkovak@fld.czu.cz (K.Š.); zeidler@fld.czu.cz (A.Z.); novakd@fld.czu.cz (D.N.); 2 Department of Sanitary and Ecological Engineering, Faculty of Civil Engineering, Czech Technical University in Prague, Thákurova 7, 160 00 Prague, Czech Republic; Jana.Nabelkova@cvut.cz; 3 Department of Materials Engineering and Chemistry, Faculty of Civil Engineering, Czech Technical University in Prague, Thákurova 7, 160 00 Prague, Czech Republic; cernyr@fsv.cvut.cz (R.Č.); klara.kobeticova@fsv.cvut.cz (K.K.)

**Keywords:** wood species effect, caffeine treatment, natural biocide, fungi, physical, mechanical properties

## Abstract

In the future, we can expect increased requirements to the health and ecological integrity of biocides used for the protection of wood against bio-attacks, and it is therefore necessary to search for and thoroughly test new active substances. Caffeine has been shown to have biocidal efficacy against wood-destroying fungi, moulds and insects. The aim of the research was to determine whether the effectiveness of caffeine, as a fungicide of natural origin, is affected by a different type of treated wood. Norway spruce mature wood (*Picea abies*), Scots pine sapwood (*Pinus sylvestris*), and European beech wood (*Fagus sylvatica*) were tested in this work. The samples were treated using long-term dipping technology or coating (according to EN 152:2012) and then tested against selected wood-destroying brown rot fungi according to the standard EN 839:2015, wood-staining fungi according to EN 152:2012, and against mould growth according to EN 15457:2015. The penetration of caffeine solution into wood depth was also evaluated using liquid extraction chromatography, as well as the effect of the treatment used on selected physical and mechanical properties of wood. The test results showed that the type of wood used and the specific type of wood-degrading agent had a significant effect on the effectiveness of caffeine protection. The most resistant wood was the treated spruce, whereas the most susceptible to deterioration was the treated white pine and beech wood. The results of the work showed that caffeine treatment is effective against wood-destroying fungi at a concentration of 2%, and at 1% in some of the tested cases. It can be used as an ecologically acceptable short-term protection alternative against wood-staining fungi in lumber warehouses and is also partially effective against moulds. It also does not have negative effects on changes in the physical and mechanical properties of the tested wood species.

## 1. Introduction

Wood products can be damaged by bio-attack during storage, transport, or assembly, but under suitable humidity conditions also in end products, e.g., wooden structures, wooden buildings, exterior and interior furniture, etc. One of the most effective ways to protect wood is the use of biocides, which prevent damage even if the structural protection is not sufficient [1]. At the same time, however, commercially used biocidal substances have negative effects on the health of workers coming into contact with them, as well as end-users, and they have overall negative environmental impacts. This, especially in industrialized countries, leads to stricter requirements for the least possible toxicity of the preparations used, in an effort to prevent the accumulation of active ingredients in the human body, animals, soil, and plants [2,3]. One method is to use substances of natural origin that have been shown not to accumulate [4,5], and caffeine is also a very affordable alternative. Its biocidal effect has been explained in several works [6,7,8]. The insecticidal effect is explained by the inhibition of phosphodiesterase activity leading to an increase in intracellular cyclic adenosine monophosphate [9]. Based on several works, the fungicidal effect of caffeine can be explained by the effect of increasing the proportion of B-glucans and chitin content with an impact on the structure of hyphal cell walls [10,11]. Tests of biocidal activity against wood-destroying fungi were initially performed by the poisoned nutrient soil methods [6], subsequently also verified on treated wood bodies [7,12]. Lightly permeable types of pine and beech wood and the method of vacuum impregnation [7,11,12] were used in most cases, but efficiency on hard-to-permeate spruce wood was also verified using long-term dipping technology [13]. Caffeine was effective against the growth of wood-destroying white and brown rot fungi [6,7,8,10,12,14] and was also tested on wooden specimens for mould growth [15]. So far, a 2% caffeine solution was tested as an effective concentration, whereas tests using lower concentrations that would reduce the cost of the used protection [1,16] are lacking. The weakness of caffeine as a natural biocide for wood protection is its washability by water [13], in which, according to research works [8,17], it only forms weak bonds. The increase in the biocidal efficiency of caffeine was investigated using subsequent heat treatment [15] and the water washability was reduced using hydrophobic treatments [12,13]. In addition to the long-term protection of built-in products, the use of caffeine as a method of short-term protection of lumber and semi-finished products is also offered, for which the biggest risk is not only the studied moulds [15] and insects [13], but also wood-staining fungi [1]. This topic has not yet been thoroughly researched. An attack by wood-staining fungi can degrade wet lumber during storage and transport under the appropriate thermal conditions within a few days [18]. Lumber degraded in this way significantly loses its price, and it is forbidden to subsequently use it for food packaging and in building structures [1].

The influence of the species of treated wood (beech and spruce) on the efficiency of caffeine has thus far been investigated in only one work [8]; otherwise, only one kind [7,11,12,13,15] was always used. However, the specific species of treated wood may have an impact on the effectiveness of the protective substance [19,20,21]. This concerns not only the quality of the impregnation [19,22], as a synergistically positive or negative effect is also possible in combination with the extractives contained in the wood, which cause its natural durability against bio-attack [8,20].

Wood is a material popularly, widely, and advantageously used in several branches of industry [3]. It is essential that any additional treatment, improving some of its original shortcomings, does not have a negative impact on its mechanical and physical properties [1,16]. Effect of caffeine treatment on wood properties changes was investigated only in a general way [13,23]. There is a lack of thorough data for this promising method of protecting wood with a natural biocide in the future.

The aim of the research was to determine whether efficacy of caffeine treatment against selected wood-destroying brown and white rot fungi, wood-staining fungi, and moulds is influenced by wood species: spruce, pine, or beech. A partial goal of the research was to compare the fungicidal effect of 1% and 2% concentration of caffeine solution, and also what effects the treatment has on selected physical and mechanical properties of the tested wood species.

## 2. Materials and Methods

### 2.1. Wood and Treatment

Norway spruce (*Picea abies*, L. Karst) mature wood, Scots pine (*Pinus sylvestris*, L.) sapwood, and European beech (*Fagus sylvatica*, L.) wood from Czech Republic was used in this experiment. The oven-dry density of spruce wood was approximately 410 kg·m^−3^, pine wood approximately 505 kg·m^−3^, and beech wood approximately 660 kg·m^−3^. Before preparing the test specimens, the timber was air-conditioned to a moisture content (MC) of ≈12%. A 1% and 2% aqueous caffeine solution (Sigma Aldrich, Prague, Czech Republic) was used to treat the test specimens, whilst only resistance to fungi was tested with the 1% treatment.

### 2.2. Mycological Tests

All mycological tests were performed in an accredited laboratory of the Timber Institute (www.vvud.cz (accessed on 5 October 2021)).

#### 2.2.1. Effectiveness of Caffeine Treatment against Rot-Fungi Attack

The test was performed in accordance with standard [24] on test specimens measuring 50 mm × 25 mm × 15 mm (L × T × R). Before applying the caffeine solution, the specimens were sealed with epoxy resin Epolex s1300 (Barvy a Laky Hostivař, Prague, Czech Republic). The treatment was then performed on the longitudinal surfaces using dipping technology. The area intake of the solution was continuously checked by weighing and it was in the range of 120 g·m^−2^ (±10 g·m^−2^) for all of the treated specimens. The test organisms were wood-destroying brown rot fungi *Coniophora puteana*, *Gloeophyllum trabeum* and *Poria (Rhodonia) placenta*. Protective efficacy was determined based on the weight loss of the wood after 16 weeks of exposure in Kole’s flasks.

#### 2.2.2. Effectiveness of Caffeine Treatment against Wood-Staining Fungi Attack

The test was performed in accordance with standard [25] on test specimens measuring 110 mm × 40 mm × 10 mm (L × T × R). Before applying the caffeine solution, the specimens were sealed with epoxy resin Epolex s1300 (Barvy a Laky Hostivař, Prague, Czech Republic). The caffeine treatment was then performed on three longitudinal surfaces using coating technology (one 110 mm × 40 mm surface was not treated). The area intake of the solution was continuously checked by weighing and it was in the range of 120 g·m^−2^ (±10 g·m^−2^) for all of the treated specimens. The test organisms were wood-staining fungi *Aureobasidium pullulans* and *Sclerophoma pithyophila*. Protective efficacy was determined after 6 weeks of exposure in cultivation containers based on visual evaluation of the specimen surfaces, where: degree 0 = no visually observable staining; 1st degree = the surface shows only isolated small areas of colouring not more than 1.5 mm wide and 4 mm long, and at no more than five locations; 2nd degree = the surface is consistently coloured up to 1/3, or locally or in strips up to half of the entire surface; 3rd degree = the surface is consistently coloured for more than 1/3, or locally for more than half of the entire surface.

The inside of the specimens after cutting was also evaluated, and the colouring of the inner surface and its extent are also monitored.

#### 2.2.3. Effectiveness of Caffeine Treatment against Mould Attack

The test was performed in accordance with standard [26] on test specimens with dimensions of 45 mm × 150 mm × 8 mm (L × T × R). The treatment was performed using dipping technology. In this case, only a 2% caffeine solution was used. The volume intake of the solution was continuously checked by weighing and it was in the range of 120 kg·m^−3^ (±10 kg·m^−3^) for all of the treated specimens. The test organisms were moulds *Penicillium brevicompactum*, *Aspergillus niger* and *Trichoderma viride*. Protective efficacy was determined after 7, 14, 21 and 28 days of fungal attack in Petri dishes based on visual evaluation of the specimens surfaces, where: degree 0 = the specimen surface is not grown over; 1st degree = the surface of the specimen is grown over up to 10% of the area; 2nd degree = the surface of the specimen is grown over between 10–30% of the area; 3rd degree = the surface of the specimen is grown over between 30–50% of the area; 4th degree = the surface of the specimens is grown over 50% of the area.

### 2.3. Physical and Mechanical Properties

Laths with surface dimensions R × T = 20 mm × 20 mm were manipulated from the central planks of beech, spruce and pine wood, with dimensions of 50 mm × 400 mm × 2000 mm (R × T × L). Test specimens (samples) with dimensions of R × T × L = 20 mm × 20 mm × 300 mm and R × T × L = 20 mm × 20 mm × 30 mm were subsequently manipulated from each lath, 3 pieces for each dimension. This number is intended to provide longitudinal parallelism for three series of experimental attempts for each monitored property (see below), thus eliminating as much as possible the factor of variability of wood structure for the possibility of the most relevant assessment of the influence of the monitored factor. The 1st sample is intended to be soaked in a 2% caffeine solution, the 2nd sample is to be soaked in distilled water, and the 3rd sample is left as a reference (without any treatment). The test specimens did not contain knots, cracks, or reaction wood, and the deflection of the fibers in the longitudinal plane was <5° [27]. Samples with a length of 30 mm were intended for density determination in oven-dry state and volume swelling, while samples with a length of 300 mm for the determination of bending parameters.

After cutting, the samples were air-conditioned until the equilibrium humidity was stabilized in the environment of the CLIMACELL 707 air-conditioning chamber (BMT Medical Technology Ltd., Brno, Czech Republic) with relative air humidity 65 ± 5% and temperature 20 ± 2 °C. Subsequently, 2 sets of samples were treated by dipping. After penetration of 120 kg·m^−3^ (±10 kg·m^−3^) caffeine or water solution, the samples were allowed to dry naturally and were then once again air-conditioned in the environment of the air-conditioning chamber at MC ≈ 12%.

The experiments for the determination of selected properties were performed according to valid harmonized standards [28,29,30,31,32] or the usual methodological procedures [33]. Laboratory scales Kern PCB 2500-2 (KERN & SOHN GmbH, Balingen, Germany), caliper Kinex 6040-27-150 (KINEX Measuring s.r.o., Prague, Czech Republic) and dryer Binder FD 115 (Binder Inc., Tuttlingen, Germany, were used to determine density and swelling. A Tira 50 kN test machine (Tira GmbH, Schalkau, Germany) was used to determine the bending parameters.

The dynamic modulus of elasticity was determined using the ultrasound method, whilst apparatus FAKOPP Ultrasound Timer UT-06/2013 (Fakopp Enterprise Bt., Ágfalva, Hungary) was used to determine the time of passage of the ultrasound wave through the test sample, followed by a computational relationship (1):

(1) $$MOE_{dyn}=c^{2}\;\cdot \;\rho \;,$$

where *MOE_dyn_* (Pa) is the dynamic modulus of elasticity, *c* (m·s^−1^) is the speed of sound propagation calculated from the time of passage of the ultrasound wave over a given length of the sample, and *ρ* (kg·m^−3^) is the wood density.

### 2.4. LC/MS Analyses

The caffeine extraction procedure was as follows: wood chips with a weight of 0.05–0.1 g were leached in 10 mL of methanol (concentration 20% *v*/*v*) overnight (24 h) and diluted in water prior to analysis (purity for HPLC) at a ratio of 1:20 and 1:200.

The caffeine concentrations were analysed on HPLC-MS/MS Agilent 1260 Infinity II (Agilent, Santa Clara, CA, USA) with Triple Quadrupole mass detection. The separation was performed through column Zorbax Eclipse Plus C18 (3 mm × 50 mm, 1.8 µm) (Agilent, Santa Clara, CA, USA) using methanol and water (acidified by formic acid 0.1:99.9, *v*/*v*) as the mobile phase in time sequence: 0–5 min 95% water, 5% methanol with 0.4 mL/min flow, 5–5.50 min 100% methanol with 0.4 mL/min flow, 5.50–5.60 min 100% methanol with 0.5 mL/min flow and 5.6–7.6 min 95% water, 5% methanol with 0.5 mL/min flow. Detection was made using Triple Quadrupole with MRM regime in positive polarity mode.

### 2.5. Statistical Evaluation

For statistical evaluation, basic descriptive statistics (mean values and standard deviations (SD)) were used, followed by an ANOVA analysis of variance to evaluate the significance of the adjustment factor. The same significance level α = 0.05 was used for the analyses. Whisker plots with means and SD, and Tukey’s HSD test at 95% significance level and all other analyses of experimental data were performed in STATISTICA Version 13.4.0.14 (TIBCO Software Inc., Palo Alto, CA, USA).

## 3. Results

**Figure 1:**
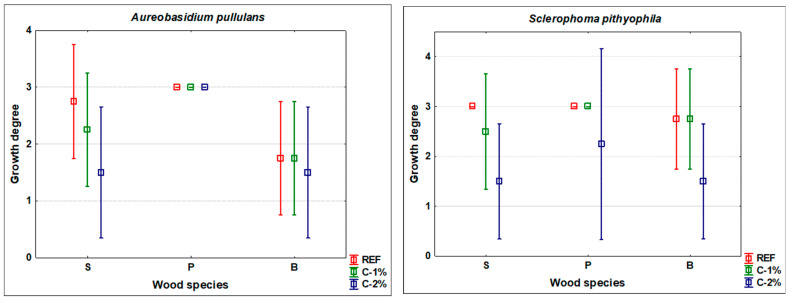
Blue-stain fungi growth.

**Figure 2:**
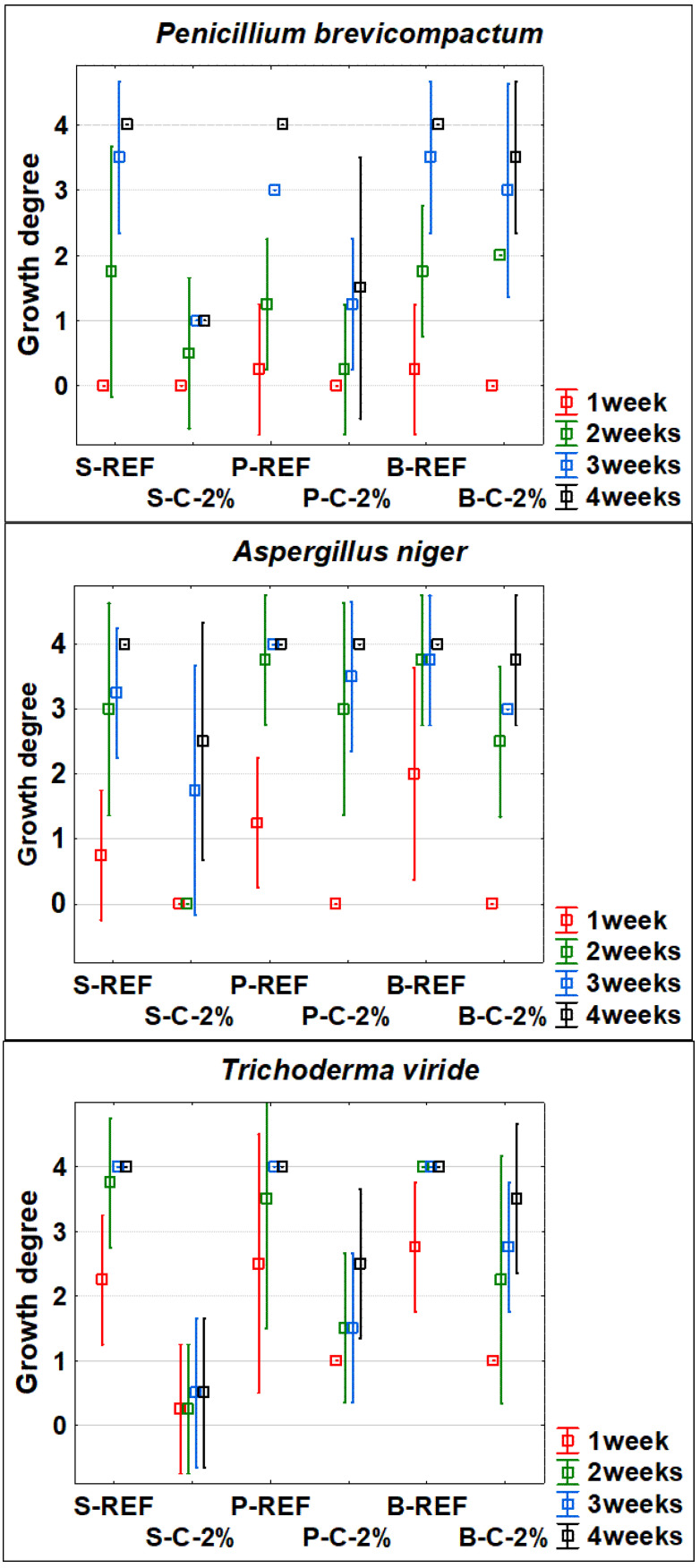
Mould growth.

**Figure 3:**
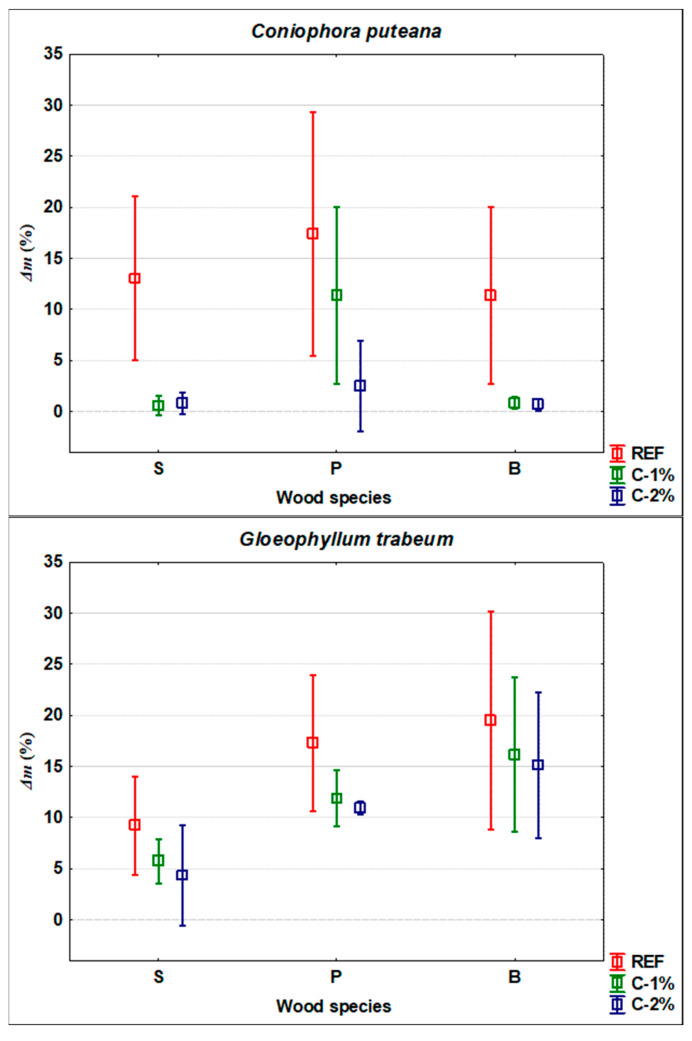

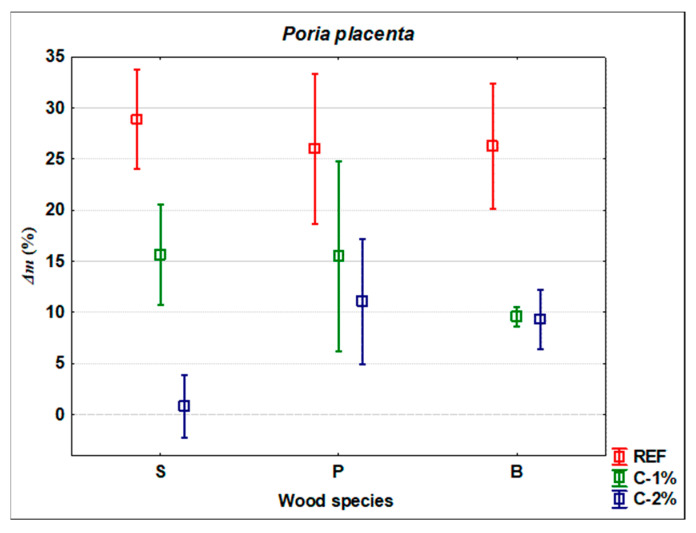
Degradation by wood-destroying fungi.

The results of the mycological tests showed a generally significant effectiveness of treatment of beech, spruce, and pine wood against fungi and mould attacks (Figure 1, Figure 2 and Figure 3). In some cases, the values achieved were relatively variable. However, even under the controlled conditions of standardized tests, a large number of factors affect the growth of living organisms [1,18].

The effect of caffeine treatment on the growth of wood-staining fungi *A. pullulans* and *S. pithyouphila* was the lowest for pine sapwood (Figure 1). Only a 2% concentration of the solution applied by coating (see Section 2.2 in Materials and Methods) was partially effective against *S. pithyophila*. The results were similar for the tested spruce and beech woods. The 1% concentration of the protective solution had a slight inhibitory effect only in spruce, whereas the 2% concentration provided a significantly more positive effect in both wood species for wood-stain mould *S. pithyophila* (Figure 1).

For the tested species of fungi, the duration of the test was a significant factor. The largest differences in the results obtained are particularly evident after 21 and 28 days of test duration (Figure 2). The influence of the type of mould and the type of tested wood was significant. The best results in inhibiting the growth of fungi on the wood surface were generally obtained with spruce. For spruce and pine woods, caffeine treatment was more effective against the growth of *P. brevi* and *T. viride*, while *A. niger* appears to be the species of mould least sensitive to the tested 2% solution treatment. For beech wood, the inhibitory effect of caffeine was observed up to a maximum of 3 weeks of test duration, but after 4 weeks there was no significant difference between treated (B-C-2%) and untreated (B-REF) wood (Figure 2).

The effect of caffeine treatment on the growth of the tested brown-rots was once again significantly influenced by the type of wood, and by the type of wood-destroying fungus (Figure 3). It was most effective against wood-destroying fungus *C. puteana*, in which, in terms of spruce and beech wood, as little as 1% of the caffeine solution concentration was highly effective. Conversely, for *P. placenta*, only spruce treated with a 2% concentration of caffeine solution achieved a weight loss of less than 3%, which is required in practice as evidence of sufficient fungicide efficacy in wood [1]. From this perspective, only the treatment of spruce is partially effective against *G. trabeum*. However, spruce wood is most often used in practice for beams and in construction that is often attacked by this type of fungus [1].

**Figure 4:**
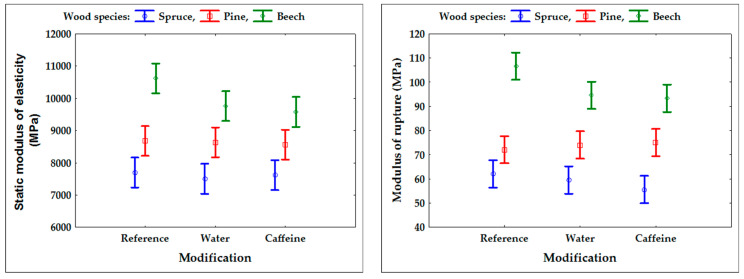
Static modulus of elasticity and modulus of rupture.

The evaluation of potential changes in selected physical and mechanical properties of the tested wood species by caffeine treatment is shown in Figure 4 and Table 1, Table 2 and Table 3. The above results clearly show that the treatment with an aqueous solution of caffeine had either no effect, or only a slight effect, on changes in the properties of the wood. The main evaluation criteria were modulus of elasticity (MOE) and modulus of rupture (MOR) (Figure 4), which react very sensitively to any damage to the wood structure [1]. The obtained values of the tested properties did not deviate from the average values and the variability reported for the given wood species [34,35]. On the other hand, due to the careful selection of the tested sets of test specimens (see Section 2.3 in Materials and Methods), the variability of the tested properties was found to be relatively low. A slight decrease in bending characteristics and volumetric swelling was only found in beech. However, the samples treated with the caffeine solution did not differ significantly from the changes caused by soaking in distilled water, and thereby the effect of caffeine can be ruled out (see Figure 4 and Table 3).

The determinations of caffeine concentrations in dipping treated samples of mature spruce wood, pine sapwood and beech wood yielded interesting findings (Table 4). The penetration of caffeine into the depth of the treated samples that had cross sections sealed with epoxy resin (see Section 2.2 in Materials and Methods) was significantly affected by the type of wood. The smallest differences in the 1st layer (0.0–0.5 mm from the wood surface) and the 2nd or the 3rd layer (0.5–1.0 mm, or 1.0–1.5 mm from the wood surface) of the depth of the layer were observed in pine sapwood, while the highest was in mature spruce and beech wood. These findings are further discussed in part 4. Another finding is that the increase in the concentration of caffeine solution from 1% to 2% applied to wood using dipping technology only led to a slight increase in the concentration of caffeine in the wood (see Table 4). From the results shown in Figure 1, Figure 2 and Figure 3, however, it is clear that these differences are crucial in many cases for the effectiveness of this treatment of wood against attack by fungi and moulds.

## 4. Discussion

The biocidal effect of caffeine was described and explained in the work of Nathason [9]. The use as a fungicide against wood-destroying fungi was first investigated in the work of Arora and Ohlan [6] and then in the work of Lekounougou et al. [10,11]. In recent years, there has been a growing interest in its use as a potentially enviro- and health-friendly wood preservative against bio-damage. Several works describing the effect on wood against wood-destroying fungi [7,8,12] have been published and the effect on termite attack has been confirmed [13].

Together with structurally similar substances, caffeine is a so-called methylxanthine. In addition to it, biocidal effects have also been investigated for theophylline and theobromine. These substances differ from each other only in the number of nitrogen atoms and their arrangement. Nevertheless, different effects of these substances against different pests were demonstrated. Caffeine is most effective against the growth of fungi, moulds, and against insect larvae. Unlike theophylline, it is persistent to the extent that it can be used for wood treatment, especially in exposures without direct effects of rainwater [8,36].

Caffeine has an inhibitory effect on fungus and moulds by interacting with the cell wall and is also capable of bioaccumulation in hyphae [37] and fertile fungi [38]. The cell wall of the fungi is responsible for the mechanical strength of cells and the fungus produces various enzymes that are involved in morphogenesis in both its formation and extinction. Such enzymes include chitinases [39]. Chemically, they are glycosyl hydrolases [40]. The groups differ from each other in amino acid sequence, 3D structure, and molecular chitinolytic reaction mechanisms [41]. The growth of fungi and moulds is thus the result of a balance between the effects of the enzymes involved in the synthesis and degradation of chitin [42] and their own self-regulation [41]. In addition, they are produced not only by fungi, but also by other organisms, including plants to protect them against fungal pathogens [42]. It can therefore be assumed that caffeine can be produced by plants precisely as an inhibitor of fungal infections. In addition, the production and inhibition of chitinases can be influenced by many factors, whilst stress regulation due to nutrient deficiency or low temperature or high osmotic pressure is also being considered [41,43].

In our work, we investigated the possible use of caffeine against wood-staining fungi, which can damage freshly felled wood in log or timber warehouses under suitable conditions within a few days [1]. The effect of the used impregnation solution concentration (1% and 2%) in combination with the type of wood (spruce, pine, beech) on mould attack, wood-destroying fungi, and wood-staining fungi was also tested for the first time. In addition, the influence of caffeine treatment on wood properties has yet to be more thoroughly documented [23,44]. This was the first testing of effectiveness against wood-destroying fungi using standard [24]: (closed test specimen faces, dipping applications), which better imitates the operating conditions of impregnation of large wood products compared to the basic test method according to [45], which has already been evaluated in other works [7,12,13].

Based on the results reported in Figure 1, Figure 2 and Figure 3, it can be stated that the type of treated wood had a relatively significant (in several cases statistically significant at the 95% level of significance) impact on the effectiveness of caffeine treatment against the tested organisms. This is in line with the results of works of more authors, where other types of fungicides were tested, but the type of wood also affected the results of weight loss or mould growth [19,20,21,46,47]. The best protective effect was achieved with spruce wood (Figure 1, Figure 2 and Figure 3). This can be explained by its higher natural durability compared to beech and pine sapwood [22]. However, Table 4, showing the caffeine concentrations in the individual layers of treated wood, offers another possible explanation. Wood-destroying and wood-staining fungi most easily attacks wood by first growing through open cell elements [1] and only then do they begin to degrade the cell wall [18]. For mature spruce wood, the tracheids are completely enclosed by pits [48] and caffeine protection on the surface of the wood and cell lumens (Table 4) provides barrier protection against hyphae penetration to greater depths. A similar concentration gradient is documented for beech wood (Table 4), which is impregnable mainly in the longitudinal direction due to open vessels [35]. However, in our test, the impregnation was mainly performed in the transverse direction, as the transverse surfaces were closed; thus, the penetration of the caffeine solution into the depth was significantly reduced (see Table 4). Since the cellular elements of beech (but also pine) are not as tightly closed as in spruce [34,35], fungal hyphae could more easily penetrate to a greater depth of the test specimens; thus, the degradations were more pronounced (Figure 1, Figure 2 and Figure 3). A better protective effect on spruce compared to beech was also achieved in the study by Kobetičová et al. [8]. This study [8] demonstrated that caffeine binds most to coumaryl alcohol (CuA) and to conipheryl alcohol (CoA), and that it interacts least with sinapyl alcohol (SA). Beech contains these components at a ratio of CoA:SA:CuA = 56:40:4 and spruce at a ratio of 94:1:5 [49] (Fengel et al., 1984). A higher ratio of SA in beech wood can lead to a lower binding of caffeine to wood; thus, its fungicidal effect can be reduced also by this reason.

The effect of the specific type of test organism used on wood damage was also confirmed (Figure 1, Figure 2 and Figure 3). This is again in line with other works [19,21] and confirmed for caffeine treatment [7,8], and in our work for an additional 3 different types of wood. The 2% concentration of caffeine treatment was most effective against the wood-destroying fungus *C. puteana*, and partially inhibiting the growth of wood-staining fungi *A. pullulans* and *S. pithiophylla* regardless of the type of wood treated. In addition, for spruce wood, it significantly inhibited the attack by wood-destroying fungi *G. trabeum* and *P. placenta*, and the growth of fungi *P.* *brevicompactum* and *T. viride*. The effect on pine sapwood and beech wood was not sufficient in a number of cases, which is in partial conflict with the works of Broda et al. [12] and Kwaśniewska-Sip et al. [7]. This is due to the use of a different method of wood treatment in our work, which may be more acceptable and cheaper for operations without the possibility of using pressure impregnation. In terms of protecting spruce wood, this procedure seems to be sufficient, which was confirmed by the work of Šimůnková et al. [13]. A treatment with 1% solution was also sufficiently effective against the attack by *C. puteana* and *G. trabeum* in spruce wood (Figure 3). However, in terms of beech and pine wood, treatment with 2% caffeine solution is only effective in some cases; it is therefore necessary to take into account the need for pressure impregnation in accordance with works Broda et al. [12] and Kwaśniewska-Sip et al. [7], with the recommendation to also confirm the results of the cited work for large-scale products used in practice.

As expected, the selected mechanical and physical properties of treated wood species were not significantly affected by treatment with an aqueous caffeine solution (Figure 4, Table 1, Table 2 and Table 3). In some cases, there was a slight (but not statistically significant) decrease in the values of bending characteristics, among which the bending strength primarily reacts sensitively to any damage to the wood structure [1,50,51]. Together with a slight decrease in volumetric swelling, this can be attributed to soaking in an aqueous caffeine or water solution (see Table 1, Table 2 and Table 3 and Figure 4), which washes out water-soluble extractives from the wood [52], and the formation of microscopic cracks can also occur during subsequent drying [53]. In our work, both of these effects only slightly disturbed the compactness of the tested specimens, and the changes in the tested properties were statistically insignificant in almost all cases. It is possible to exclude the concurrent effect of the caffeine solution on the observed changes, as the effects of pure water had the same effect (Table 1, Table 2 and Table 3 and Figure 4). For beech wood, the slightly more pronounced effect of soaking in aqueous caffeine and in water compared to coniferous species is associated with the significantly different wood structure and transport processes in the beech wood [23,54,55].

## 5. Conclusions

Caffeine appears to be an ecological, safe, and affordable alternative to conventional biocides. In our work we tested the effect of 1% and 2% concentration of aqueous caffeine solution applied via dipping on spruce, white pine, and beech woods against brown rot wood-destroying fungi *Coniophora puteana*, *Gloeophyllum trabeum,* and *Poria (Rhodonia) placenta*. The coating application was tested against wood-staining fungi *Aureobasidium pullulans* and *Sclerophoma pithyophila*. A 2% solution was tested by dipping against the growth of moulds *Penicillium brevicompactum*, *Aspergillus niger* and *Trichoderma viride*.

The test results showed that the type of wood used and the specific type of wood degrading agent had a significant effect on the effectiveness of caffeine protection. The treated spruce wood was most resistant to damage by the tested organisms, while the most susceptible to deterioration was the treated white pine and beech wood. The results of the work showed that the caffeine treatment is effective against wood-destroying fungi at a concentration of 2%, and at 1% only in some of the tested cases. It can be used as an ecologically acceptable short-term protection alternative against wood-staining fungi in lumber warehouses, and the 2% concentration is also partly effective against the growth of mould on wood. It also does not have negative effects on changes in the physical and mechanical properties of the tested wood species. Treatment of wood with caffeine is also suitable for practical purposes. In particular, it can preventively protect wood against bio-attack, if the final product is exposed to accidental moisture or condensed water.

## Figures and Tables

**Table 1:** Basic statistical analyses, mean value (standard deviation) of the selected properties for spruce wood.

Modification	Density (kg·m^−3^)	Oven-Dry Density (kg·m^−3^)	Volumetric Swelling (%)	Dynamic MOE (MPa)
Reference	434	411	14.9	12,703
	(16)	(14)	(0.7)	(1427)
Water	444	406	14.4	13,152
	(17)	(10)	(1.0)	(2060)
Caffeine	449	412	14.6	12,864
	(18)	(13)	(1.2)	(1893)

**Table 2:** Basic statistical analyses, mean value (standard deviation) of the selected properties for pine wood.

Modification	Density (kg·m^−3^)	Oven-Dry Density (kg·m^−3^)	Volumetric Swelling (%)	Dynamic MOE (MPa)
Reference	566	505	13.4	14,134
	(13)	(11)	(1.6)	(2137)
Water	573	500	13.1	14,154
	(24)	(12)	(1.9)	(1555)
Caffeine	574	515	13.0	13,068
	(10)	(11)	(2.1)	(1113)

**Table 3:** Basic statistical analyses, mean value (standard deviation) of the selected properties for beech wood.

Modification	Density (kg·m^−3^)	Oven-Dry Density (kg·m^−3^)	Volumetric Swelling (%)	Dynamic MOE (MPa)
Reference	691	662	23.3	16,398
	(13)	(12)	(0.8)	(1010)
Water	689	657	22.4	16,481
	(10)	(9)	(1.1)	(1050)
Caffeine	680	653	21.5	15,384
	(19)	(8)	(0.5)	(1483)

**Table 4:** Concentrations of caffeine (mg of caffeine/g of wood) in 3 layers of caffeine treated spruce, pine and beech samples.

Concentration of Caffeine Solution	Depth of Treated Wood Layer	Concentration of Caffeine in Wood (mg of Caffeine/g of Wood)		
		Mature Spruce Wood	Pine Sapwood	Beech Wood
c = 1%	1st layer (0.0–0.5 mm)	9.31 (0.29)	9.13 (0.52)	5.50 (0.18)
	2nd layer (0.5–1.0 mm)	1.39 (0.38)	7.77 (1.05)	1.02 (0.10)
	3rd layer (1.0–1.5 mm)	0.79 (0.24)	5.04 (0.11)	0.80 (0.02)
c = 2%	1st layer (0.0–0.5 mm)	11.49 (0.22)	9.95 (1.97)	8.85 (0.53)
	2nd layer (0.5–1.0 mm)	0.52 (0.18)	6.48 (0.33)	1.30 (0.17)
	3rd layer (1.0–1.5 mm)	0.61 (0.11)	5.15 (0.03)	1.36 (0.35)

## Data Availability

Not applicable.
